# Outcomes of Atrioseptostomy with Stenting in Patients with Pulmonary Arterial Hypertension from a Large Single-Institution Cohort

**DOI:** 10.3390/diagnostics10090725

**Published:** 2020-09-21

**Authors:** Sergey V. Gorbachevsky, Anton A. Shmalts, Gulomjon M. Dadabaev, Nasirullo A. Nishonov, Manolis G. Pursanov, Vladimir A. Shvartz, Sergey B. Zaets

**Affiliations:** 1 Department of Pulmonary Hypertension, A.N. Bakoulev National Medical Research Center of Cardiovascular Surgery, 135 Roublevskoye Shosse, 121552 Moscow, Russia; sgorbachevsky@bakulev.ru (S.V.G.); shmaltzanton@inbox.ru (A.A.S.); dgmed83_83@mail.ru (G.M.D.); dr.naro@mail.ru (N.A.N.); mpursanov@rambler.ru (M.G.P.); shvartz.va@ya.ru (V.A.S.); 2 Retired from A.N. Bakoulev National Medical Research Center of Cardiovascular Surgery, 135 Roublevskoye Shosse, 121552 Moscow, Russia

**Keywords:** catheters, lung diseases, lung transplantation, pulmonary circulation

## Abstract

The aim of this study was to analyze results of stenting atrioseptostomy in patients with pulmonary arterial hypertension and a different level of risk for one-year mortality that is not well described. Patients that underwent atrioseptostomy with stenting were retrospectively divided in two groups: “intermediate” (*n* = 55) or “high” risk (*n* = 13), according to the 2015 ESC/ESR guideline. Results of atrioseptostomy were assessed during hospital period and at follow-up. Patients from “intermediate” risk group demonstrated lower mortality rate (10/55, vs. 6/13) during the course of the study period, as well as higher freedom from lung transplantation or Potts shunt. At discharge, patients of both groups presented improvement in functional class and mobility. Patients from “intermediate” risk group showed longer 6-min walking distance, and lower levels of brain natriuretic peptide. At the latest follow-up, stable position and full patency of stents with right-to-left or bidirectional shunt at atrial level and absence of syncope was confirmed in patients of both groups. Patients from the “intermediate” risk group demonstrated higher functional class, better performance of walking test, and lower levels of brain natriuretic peptide. Stenting atrioseptostomy reliably secured interatrial communication and improved clinical condition in patients with idiopathic pulmonary arterial hypertension. Mid-term results were better in “intermediate” risk group.

## 1. Introduction

Idiopathic pulmonary arterial hypertension (PAH) is a severe disease that often quickly progresses and leads to fatal outcomes. Rising pulmonary vascular resistance results in increasing systolic overload of the right cardiac chambers and congestive heart failure, as well as in insufficient blood flow to the left cardiac chambers and decreased cardiac output. PAH-specific drug therapy allows to improve patients’ prognosis and life expectancy. Methods of choice for drug-refractory PAH include atrial septostomy (AS), Potts shunt, and lung transplantation. It should be also mentioned that full spectrum of PAH-specific therapy may not be available in certain countries.

According to current PAH treatment recommendations, AS can be considered for patients in III–IV functional class with syncope and/or severe right ventricular failure that is refractory to maximal drug therapy, or if this therapy is not available [1]. The main rationale for AS in PAH is creating and maintaining atrial septal communication with right-to-left shunting that can achieve decompression of the right cardiac chambers, as well as increase the preload of the left chambers, but with no development of severe hypoxemia. This intervention improves the patients’ condition. However, results of atrial septostomy in patients with different levels of risk are not well described.

Over the years, several methods of AS have been suggested. All of them have certain limitations or disadvantages. Initially commonly used, blade AS frequently resulted in severe hypoxemia and poor outcomes because of the uncontrolled size of created atrial septal communication [2,3,4]. The introduction of graded stepwise balloon AS allowed to minimize risks of “insufficient” or “excessive” atrial septal communication. However, its frequent spontaneous closure at late follow-up led to the necessity of repeat interventions in this critically-ill patient population [5,6,7,8]. The implantation of various devices may potentially secure necessary diameter of atrial septal communication for a long period of time [9,10]. However, this technique cannot be considered to be a well-established procedure because of controversial results and relatively small clinical experience. Experience with stent fenestratration of the atrial septum is very limited and does not exceed 15 interventions at one particular institution [11,12,13,14,15,16,17] (Table 1).

The aim of this work was to analyze results of AS with stenting performed in patients with idiopathic PAH of different risk levels.

## 2. Materials and Methods

### 2.1. Regulatory Aspects

The protocol of this retrospective medical chart review study was approved by the Institutional Review Board of the A.N. Bakoulev National Medical Research Center of Cardiovascular Surgery (No. 2019-03-012 from 3rd March 2019) with a waiver of consent. However, all patients’ parents/legal guardians did provide written consent at admission that allowed using the data from their charts received during pre-operative examination, surgical intervention, and follow-up for scientific analysis and publication.

### 2.2. Patient Population

In the years 2006–2019, sixty-eight patients with idiopathic PAH underwent 68 procedures of AS with stenting. Pre-operative assessment included collection of historical data, demographic information, and physical examination with the determination of World Health Organization (WHO) functional class. Pre-operatively, all patients were subjected to echocardiographic examination and cardiac catheterization. Pre-operative six-minute walk distance (6MWD) test with SaO2 measurement was possible in all patients, except two in critical condition, and one child below 6 years of age. Post-operative 6MWD test was performed at discharge in all patients, except the aforementioned child. The level of brain natriuretic peptide was measured at admission and at discharge.

Fifty-five patients were followed-up after discharge (individual data included to the analysis represent the latest examination). In all cases, assessments included physical examination, 6MWD test with SaO2 measurement, echocardiography, and brain natriuretic peptide measurement. Twenty-three patients were subjected to cardiac catheterization.

For retrospective analysis, patients were divided into two groups, according to 2015 Guidelines for the Diagnosis and Treatment of Pulmonary Hypertension [1]: Group 1—“intermediate risk” (5–10%) for one-year mortality (*n* = 55), and Group 2—“high risk” (> 10%) for one-year mortality (*n* = 13).

### 2.3. Surgical Technique

Interventions were performed under general anesthesia with endotracheal intubation. The procedure was monitored via transesophageal echocardiography. Access to the atrial septum was achieved via the right femoral vein. Heparin was routinely infused after trans-septal puncture. For the first 5 cases, peripheral vascular stents Palmaz–Genezis (Cordis, Santa Clara, CA, USA) or Hippocampus (Medtronic, Minneapolis, MN, USA) were used. Their diameter in the narrowest area and length ranged from 4 to 8 mm and from 20 to 30 mm, respectively. For the remaining 63 cases, Palmaz–Genezis 2910 or Palmaz–Genezis 3901 stents of bigger diameter with length of 29 or 39 mm were modified to obtain a “diabolo” shape, using the loop made from epicardial electrode (4–8 mm in diameter), according to the method described by Stumper et al. [11]. Stents were mounted on 14–16 mm maxi LD balloon catheter (Cordis, USA). Stents were positioned the way to put the loop exactly to the middle of atrial septum. During implantation, ends of the stent were opened to the desired diameter, and the middle of the stent to the diameter of the loop. Eventually, stents received a “diabolo” shape. The diameter of the created atrial communication was 4 mm in pediatric patients and 6–8 mm in adults.

### 2.4. Statistical Analysis

Statistical analysis was performed using STATISTICA version 10.0 (StatSoft, Tulsa, OK, USA) software. Variables were checked for normality using the Shapiro–Wilk test and presented as appropriate: continuous as medians and interquartile range (Q1–Q3), and categorical as absolute numbers and proportions (%). Continuous variables were compared with the Mann–Whitney U-test or the Wilcoxon signed-rank test as appropriate. Categorical variables were compared via the Fisher’s exact test. Kaplan–Meier survival and freedom from lung transplantation or Potts shunt were determined for each group and compared by the log-rank test. *p* values less than 0.05 were considered statistically significant.

## 3. Results

Age of patients as well as time from diagnosis of PAH and until AS did not significantly differ in compared groups (Table 2). Clinical signs of right heart failure were recorded in all patients of the “high risk” group. Liver enlargement, peripheral edema, ascites, and jugular venous distension were diagnosed in 13 (100%), 10 (77%), 2 (15%), and 1 (7.5%), respectively. The majority of patients in the “intermediate risk” group did not have manifestation of right heart failure. Symptoms were progressing rapidly (within 6 months) in the “high risk” group, whereas in the “intermediate risk” group, the progression of symptoms was relatively slow (within 2 years). Not surprisingly, patients in the “high risk” group were in a worse WHO functional class and demonstrated inferior performance of 6MWD test, as well as significantly lower SaO2 after the test (Table 2). Peak VO_2_ during cardiopulmonary exercise test was significantly lower in patients from the “high risk” group: 10.6 (10.2–10.9) vs. 14.1 (13.2–15.0) ml/min/kg, (*p* < 0.001). They also had significantly higher values of brain natriuretic peptide, as well as a higher degree of tricuspid regurgitation and atrial pressure (Table 2). Right atrial area per echocardiography was significantly larger in the “high risk” group: 27.5 (25.9–29.0) vs. 21.4 (18.3–24.7) cm^2^, (*p* < 0.001). Pericardial effusion exceeding 5 mm per echocardiography was diagnosed in all patients from the “high risk” group. On the contrary, patients in the “intermediate risk” group used to have either minimal (≤ 5 mm) or no pericardial effusion (in 10 (18%) or 45 (82%) cases, respectively). Patients from the “high risk” group also demonstrated significantly lower hemodynamic indexes (cardiac index and SvO_2_): 1.94 (1.56–2.05) vs. 2.2 (2.05–2.4) l/min/m^2^, (*p* < 0.001) and 58 (57–60) vs. 62 (60–63) %, (*p* < 0.001), respectively.

PAH-specific therapy was indicated for all patients in both groups. However, it was available for only 29 (53%) patients in Group 1 and 7 (54%) patients in Group 2 because of various financial and/or organizational reasons. It should also be mentioned that intravenous epoprostenol could not be administered, because this medication is not licensed in the Russian Federation. Monotherapy, two- or three-component combined PAH-specific oral therapy included bosentan, sildenafil and/or inhalation of iloprost. Two patients from Group 2 were admitted into the intensive care unit prior to intervention for oxygen inhalation and cardiotonic support. Indication for AS was syncope and/or refractory severe right ventricular failure.

The AS procedure was uneventful in 66 patients. In two cases, the loop was misplaced to the distal end of the balloon catheter that caused the necessity of bringing the delivery system down to the inferior venae cava, to implant the stent there, and to use another stent to secure patency of created atrial communication. Measurement of hemodynamic parameters in the operating room immediately after AS demonstrated that right atrial pressure in both groups had significantly decreased, whereas left atrial pressure significantly increased (Table 2). However, absolute values of atrial pressure remained significantly higher in the “high risk” group.

All patients survived intervention and were transferred to the Intensive Care Unit (ICU). Duration of mechanical ventilation in the “intermediate” and “high” risk group (2.8 (1.4–4) and 3 (2–4) hours, respectively) did not significantly differ (*p* = 0.295). After extubating in the ICU, all patients were receiving inhalation with oxygen, nitric oxide, and iloprost, as well as PAH-specific oral therapy. Three patients from the “high risk” group died of repeated episodes of pulmonary hypertensive crisis on the 2nd, 10th, and 12th day after intervention, respectively. Two more patients (one in each group) experienced non-lethal pulmonary arterial crisis during the hospital stay. None of the patients experienced syncope during the hospital period.

Examination at discharge revealed that WHO functional class had significantly improved in both groups, if compared to pre-operative values (Table 2). However, it remained significantly worse in the “high” than in the “intermediate” risk group. Patents of both groups demonstrated significantly increased 6MWD, compared to pre-operative values (absolute values remained significantly lower in the “high” than in the “intermediate” risk group). SaO2 at rest as well as after exercise has decreased post-intervention in both groups. However, none of the patients experienced severe hypoxemia. In both groups, SaO2 was decreasing after the walking test. Pre- and post-test SaO2 level was significantly lower in the “high” than in the “intermediate” risk group. As per the transthoracic echocardiography at rest, all patients in the “high risk” group and 52 out of 55 patients in the “intermediate risk” group demonstrated right-to-left or bidirectional shunt at atrial communication. In three patients from the “intermediate risk” group, a left-to-right shunt was present. After the 6MW test, a right-to-left or a bidirectional shunt was recorded in all patients. Tricuspid regurgitation has significantly decreased after intervention in both groups and no longer differed between groups. Brain natriuretic peptide (BNP) levels have significantly dropped after AS, but remained higher in “high” risk group.

**Figure 1:**
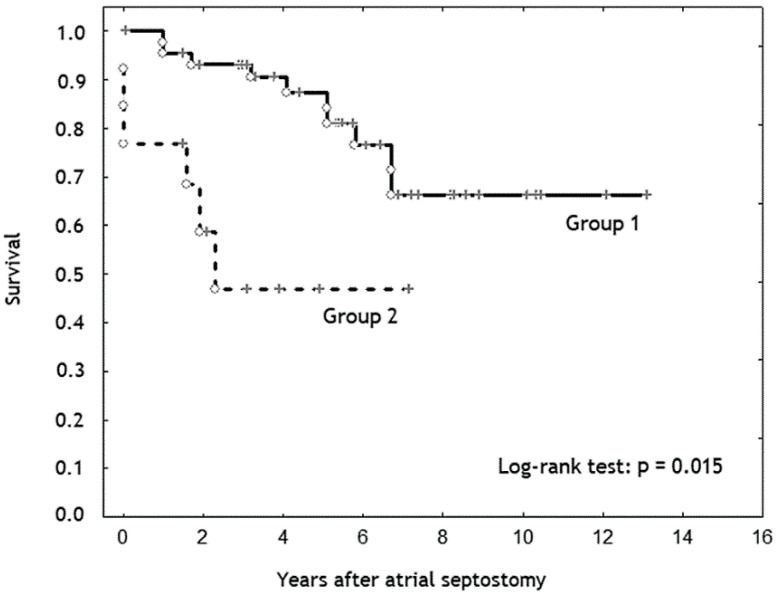
Kaplan–Meier survival curve. Patients from the “intermediate” risk group (Group 1) demonstrate significantly higher survival rate than patients from the “high” risk group (Group 2) (*p* = 0.015).

**Figure 2:**
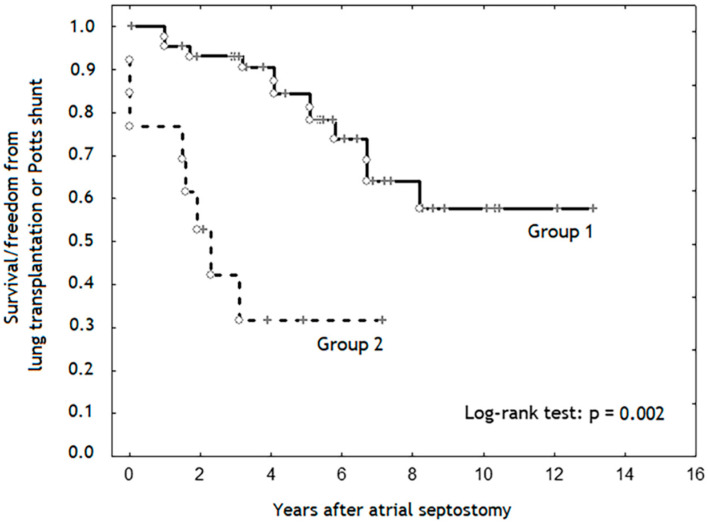
Kaplan–Meier survival and freedom from lung transplantation or Potts shunt curve. Patients from the “intermediate” risk group (Group 1) demonstrate significantly higher survival rate/freedom from lung transplantation or Potts shunt than patients from the “high” risk group (Group 2) (*p* = 0.002).

The duration of follow-up was significantly longer in the “intermediate” than in the “high” risk group: 5.4 (3.3–7.3) vs. 2.2 (1.6–4) years, (*p* < 0.001). Patients from the “intermediate” risk group demonstrated significantly higher survival rate and freedom from lung transplantation or Potts shunt than in the “high” risk group (Figure 1 and Figure 2).

There were 13 lethal outcomes (10 in “intermediate” and 3 in “high” risk group) during follow-up period. In the majority of cases, death was caused by progressive PAH (Table 3 presents causes of lethal incomes during the entire course of the study).

**Figure 3:**
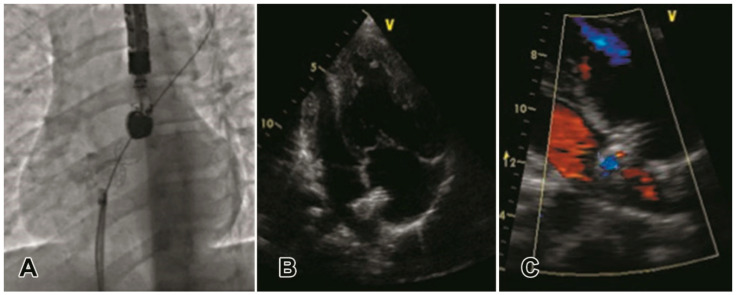
Examination after AS with stenting. (**A**—fluoroscopy immediately after stenting; **B**, **C**—transthoracic echocardiography at follow-up). Stent is located correctly. Right heart chambers are enlarged; right-to-left shunt across the atrial septum is visualized.

**Figure 4:**
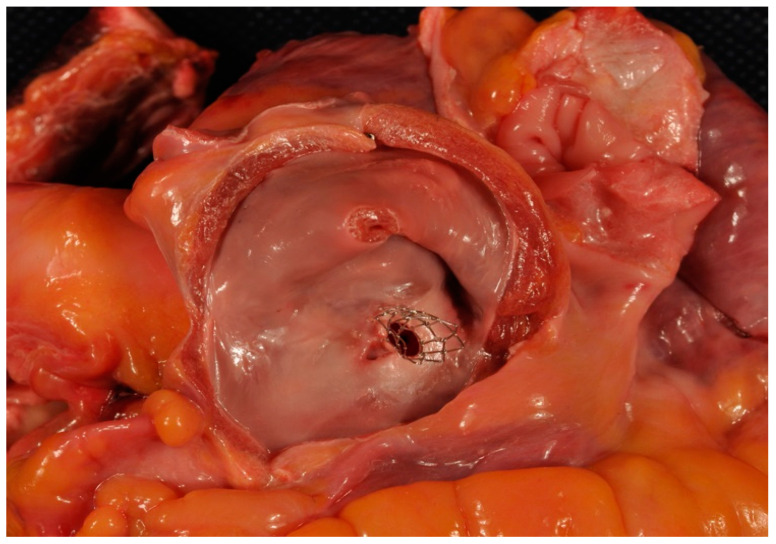
Autopsy of the patient that died of pulmonary hypertensive crisis developed after cardiac catherization at 5 years after AS. Left atrium is opened. Full patent stent is seen.

One patient from the “intermediate” risk group experienced ischemic stroke with a gradual regress of neurological symptoms. Deterioration of clinical condition caused the necessity of Potts shunt in one patient from the “high” risk group and lung transplantation in two patients (one in each group). All patients in both groups were receiving anticoagulants (warfarin, rivaroxaban or dabigatran etexilate) and/or antiplatelet medications (aspirin). During the period of follow-up, one-component, two- or three-component combined PAH-specific oral therapy, that included bosentan, macitentan, ambrisentam, sildenafil, riociguat, and/or inhalation of iloprost, became available for all 10 followed-up patients in the “high” risk group and for 43 of 45 (95.5%) followed-up patients in the “intermediate” risk group. A WHO functional class at the latest examination in the “intermediate” risk group was better than in the “high” risk group: 3.0 (2.0–3.0) vs. 4.0 (3.0–4.0), respectively, (*p* = 0.002). None of the patients complained on syncope. Patients from the “intermediate” risk group continued demonstrating better performance of 6MW test [367 (298–414) vs. 215 (171–295)] m, *p* = 0.006] as well as lower levels of BNP [118 (88–170) vs. 210 (196–454) pg/mL, *p* = 0.008] compared to the “high” risk group. SaO2 at rest and after the walking test did not differ in the compared groups [88 (86–91) in the “intermediate” risk group vs. 86 (85–88) % in the “high” risk group, *p* = 0.204; and 78.5 (74.5–81.5) vs. 76 (75–77) %, *p* = 0.205, respectively]. Echocardiographic parameters (systolic right ventricular pressure and tricuspid regurgitation) also did not differ in the compared groups [105 (97–125) in the “intermediate” risk group vs. 110 (95–135) mm Hg in the “high” risk group, *p* = 0.718; and 2 (1.5–3) vs. 3 (2.5–3), *p* = 0.087, respectively]. All patients demonstrated right-to-left or bidirectional shunt at atrial communication at rest, as well as after 6MW test. Because only two patients in the “high risk” group underwent cardiac catheterization at follow-up, we were unable to compare hemodynamic parameters between groups. Stable position and full patency of stents were confirmed in all cases without exception at follow-up examination (Figure 3) or autopsy (Figure 4).

## 4. Discussion

More than 35 years have passed since Rich S and Lam W performed the first AS for refractory PAH [18]. This concept was based on experimental studies using a dog model of PAH and demonstrating that right-to-left shunting increases cardiac index [19]. Later on, clinical observational studies have confirmed the correctness of the aforementioned concept, showing that patients with Eisenmenger’s syndrome have better cardiac performance and prognosis than those with PAH [20], and that concomitant interatrial communication in patients with PAH contributes to longer survival [21,22]. It has been objectively proven during these three decades that AS at least temporarily improves patients’ condition, eliminates congestive heart failure and syncopes as well as serves as a “bridge” to lung transplantation [6,9,13,23]. Our findings clearly support these conclusions. All patients that survived the hospital period demonstrated an improvement in functional class and increase in 6MWD already at discharge. None of them reported syncopes. There was no severe hypoxemia. The most frequent early complication was pulmonary hypertensive crisis that led to lethal outcomes in three patients. We did not observe any clinically relevant arrhythmias reported by others [10,16,23]. It is also known that AS does not stop the progression of the pulmonary vascular disease, and it is this progression that has caused 77% of lethal outcomes during the follow-up in our patient population. However, the AS temporarily improves patients’ condition and gives them time to find additional opportunities, such as access to modern approved PAH-specific therapy if this was not previously available, or lung transplantation.

At the same time, certain aspects related to AS performed in patients with PAH are still disputable. First, the preferable method of AS has not yet been established. The initially used blade AS has been eventually abandoned because of uncontrolled size of created interatrial communication that caused severe hypoxemia, leading to lethal outcomes [2,3]. Graded stepwise balloon AS is free from this limitation, because the size of fenestration performed under hemodynamic control can be determined individually for each particular patient. However, even in a relatively large series of cases, 18–39% of patients that survived hospital period, require repeat intervention because of spontaneous closure of interatrial communication [6,7,23]. It is difficult to assess effectiveness of fenestrated occluders that have been used during the last decade. This is mainly because the number of procedures at one particular institution does not exceed 12, and the maximum duration of follow-up is short (no longer than 31 months) [10,24]. Besides, data on spontaneous closure of fenestration are contradictory. Rajeshkumar R et al. reported the patency of all implanted devices [10], whereas in the series described by Lammers AE et al., 44% of survivors experienced the spontaneous closure of the custom-made fenestrated Amplatzer device [24]. Potential disadvantages of fenestrated devices include the implantation of a substantial amount of foreign material into the atrium in this vulnerable patient population, as well as the technical impossibility of enlarging interatrial communication if clinically needed. World experience of AS with stenting is also relatively small (the largest published series includes 15 cases with the maximum period of follow-up equal to 5.9 years) [13] (Table 1). In our series of 68 interventions, stents were patent and remained in a stable position in all patients that were followed-up for a maximum period of 13 years after intervention. This corresponds to available literature data that do not report a single case of stent fenestration closure.

Indications for AS in patients with PAH also require a more detailed assessment. ESC/ERS guidelines for the diagnosis and treatment of pulmonary hypertension [1] consider AS appropriate in patients “in WHO III-IV functional class with right heart failure refractory to optimal medical therapy or with severe syncopal symptoms [5,25]”. Intervention “may also be considered in patients awaiting lung transplantation with unsatisfactory clinical response to maximal medical therapy or when medical therapy is not available”. At the same time, some authors prefer the earlier performance of AS in infants, children, and adolescents. In the series presented by Sandoval J et al., 62% of patients were not receiving PAH-specific therapy prior to AS [6]. Authors do not suggest postponing the intervention until medical therapy becomes ineffective. They hypothesize that intervention performed “at a relatively early stage of the disease” may achieve promising results. Authors were adding pharmacological therapy only after AS if it was available, and came to a conclusion that this strategy is more effective than the AS alone. In our retrospective series, only approximately half of patients had access to PAH-specific therapy prior to the AS. This situation has dramatically changed during the period of follow-up post-AS, when the vast majority of patients gradually received an opportunity for necessary treatments.

Contraindications to AS include end-stage disease with right atrial pressure >20 mm Hg and O2 saturation <85% on room air [1]. Multiple authors reported high peri-procedural mortality in this category of patients [5,7,15].

One of our aims was to analyze the results of AS performed in patients with different levels of risk. This analysis has demonstrated that early lethal outcomes occurred only in the group of patients with “high” risk for one-year mortality, per ESC/ERS guidelines for the diagnosis and treatment of pulmonary hypertension [1]. Patients with “high” risk used to have lower survival rates and freedom from lung transplantation or Potts shunt than patients with “intermediate” level of risk.

Our work has at least several limitations. First of all, this is a retrospective study with no control group and randomization that could better prevent any bias. Furthermore, the number of patients in compared groups with a different level of risk is unequal. Finally, the duration of follow-up is relatively short and significantly differs between the groups.

## 5. Conclusions

We can conclude that AS with stenting in patients with PAH is a safe procedure with a low hospital mortality. It allows one to create interatrial communication of a given diameter that does not cause clinically relevant hypoxemia, remains patent in at least mid-term perspective, and does not require repeat interventions. AS with stenting achieves significant hemodynamic and clinical improvement and may serve as a “bridge” not only to Potts shunt or eventually to lung transplantation, but also to modern approved PAH-specific drug therapy if it is not routinely available pre-intervention. The course of the hospital period was more favorable in patients with “intermediate” risk. They also demonstrated better survival and freedom from lung transplantation or Potts shunt. However, advantages of AS performed in the “intermediate” risk patient category should be confirmed by controlled randomized studies.

## Figures and Tables

**Table 1:** Atrial septostomy with stents in patients with pulmonary arterial hypertension (PAH): world experience.

Authors	Number of Cases	Age at Intervention (Years)	Type of Stents	Perioperative/30-Day Mortality (%)	Duration of Follow-Up, Outcome, Surgical Procedures, Stent Patency
Stümper, O. et al. 2003 [11]	6	7.5–72	Standard stent (Johnson & Johnston P308 or P188, or Jomed 17-mm), diabolo-shaped configuration	No	Median—1.05 (max—2.1) years. One death. One heart-lung transplantation. All stents were patent.
Prieto, L.R. et al. 2006 [12]	1	32	Palmaz-Schatz 18-mm stent (Johnson & Johnson), butterfly-shaped configuration	No	4 months. Lung transplantation. Stent was patent.
Troost, E. et al. 2009 [13]	15	48.2 ± 20.5	Palmaz Genesis 1910 stent (Cordis Corporation), diabolo-shaped configuration	1 (6.5%)/2 (13%)	Medium—0.7 (max—5.9) years. Six deaths. Four lung transplantations with 2 deathsAll stents were patent.
Roy, A.K. et al. 2013 [14]	1	68	Palmaz Genesis 10 × 29 mm peripheral stent (Cordis Corporation), butterfly-shaped configuration	No	1 year. Stent was patent.
Kuhn, B.T. et al. 2015 [15]	6	Not reported specifically for patients with PAH	7 × 18 mm peripheral stent	No	Not reported specifically for patients with PAH. All stents were patent.
Velazquez Martín, M et al. 2016 [16]	1	64	Palmaz Genesis 19-mm stent (Johnson & Johnson), diabolo-shaped configuration	No	44 months. Stent was patent.
Degano Iglesias, L.A. et al. 2019 [17]	9	0.38–11.8	Palmaz Genesis 10 × 19 mm stent, diabolo-shaped configuration	No	Median—25.85 months (including 2 patients with non-stenting techniques). Two deaths. Three lung transplantations. Stent patency is not described.

PAH—pulmonary arterial hypertension.

**Table 2:** Clinical and hemodynamic parameters before and at discharge after atrial septostomy.

	Before AS			After AS			*p* Before/After AS Group 1	*p* Before/After AS Group 2
	Group 1 (*n* = 55)	Group 2 (*n* = 13)	P	Group 1 (*n* = 55)	Group 2 (*n* = 10)	P		
Age (years)	20 (12–34)	32 (13–37)	0.198	N/A	N/A	N/A	N/A	N/A
Time from PAH diagnosis and AS (months)	10 (5–16)	7 (5–18)	0.708	N/A	N/A	N/A	N/A	N/A
WHO functional class	3 (3–3)	4 (4–4)	<0.001	2 (2–3)	3 (3–3)	0.005	<0.001	0.005
Syncope	42 (76%)	9 (69%)	0.690	0 (0%)	0 (0%)	1.0	<0.001	<0.001
SaO_2_ at rest (%)	97 (95–98)	95 (94–97)	0.059	91 (89–93)	89 (88–90)	0.010	<0.001	0.024
6MWD (m)	397 (334–432)	198 (134–284)	<0.001	423 (388–469)	299 (240–357)	<0.001	<0.001	0.015
SaO_2_ after 6MWD test (%)	96 (93–97)	92 (90–93)	0.003	83 (81–87)	82 (79–82)	0.036	<0.001	0.010
BNP (pg/mL)	203 (98–400)	854 (399–1246)	0.002	86 (48–134)	217 (97–389)	0.023	<0.001	0.007
**Echocardiography**								
RV systolic pressure (mm Hg)	100 (90–120)	110 (100–120)	0.399	100 (90–120)	100 (84–110)	0.389	0.250	0.192
Tricuspid regurgitation	2 (1.5–3)	3 (2.5–3)	0.042	2 (1.5–2)	2 (1.5–2.5)	0.512	<0.001	0.027
**Cardiac Catheterization or Intraoperative Measurement**								
RA pressure (mm Hg)	14 (11–16)	16 (15–19)	0.003	11 (10–14) *	14 (13–16) *	0.008	<0.001	0.002
LA pressure (mm Hg)	6 (5–8)	9 (8–11)	0.008	8 (7–9) *	11.5 (10.5–13) *	0.001	<0.001	0.002
Mean PA pressure (mm Hg)	64 (54–81)	62 (58–73)	0.668	71 (61–87) *	73 (61–83) *	0.812	0.001	0.173
Mean PA pressure/Mean arterial pressure	0.81 (0.66–1.05)	0.78 (0.60–0.91)	0.522	0.90 (0.70–1.09) *	0.98 (0.73–1.11) *	0.557	0.035	0.066

* Measurement was performed at intervention upon completion of atrial septostomy. AS—atrial septostomy; BNP—brain natriuretic peptide; LA—left atrium; PA—pulmonary artery; PAH—pulmonary arterial hypertension; RA—right atrium; RV—right ventricle; WHO—World Health Organization; 6MWD—six-minute walk distance.

**Table 3:** Causes of lethal outcomes after atrial septostomy.

Patient Number/Time after Atrial Septostomy	Cause of Lethal Outcome
Group 1 (“intermediate risk”)	
1. One year	Progressive PAH
2. One year	Pulmonary hypertensive crisis after appendectomy
3. One year and 8 months	Pulmonary hypertensive crisis after teeth extraction
4. Three years and 2 months	Progressive PAH
5. Four years and 1 months	Progressive PAH
6. Five years	Pulmonary hypertensive crisis after cardiac catheterization
7. Five years and 1 month	Progressive PAH
8. Five years and 10 months	Progressive PAH
9. Six years and 8 months	Progressive PAH
10. Six years and 8 months	Progressive PAH
Group 2 (“high risk”)	
1. Second day	Repeated pulmonary hypertensive crisis
2. Tenth day	Repeated pulmonary hypertensive crisis
3. Twelfth day	Repeated pulmonary hypertensive crisis
4. One year and 7 months	Progressive PAH
5. One year and 11 months	Progressive PAH
6. Two years and 4 months	Progressive PAH

PAH—pulmonary arterial hypertension.
